# Treatment and survival patterns of Chinese patients diagnosed with breast cancer between 2005 and 2009 in Southwest China: An observational, population-based cohort study

**DOI:** 10.1097/MD.0000000000003865

**Published:** 2016-06-24

**Authors:** Zuxiang Peng, Jia Wei, Xuesong Lu, Hong Zheng, Xiaorong Zhong, Weiguo Gao, Yunqin Chen, Jing Jing

**Affiliations:** a Department of Thyroid and Breast Surgery, Laboratory of Molecular Diagnosis of Cancer, State Key Laboratory of Biotherapy, National Collaborative Innovation Center for Biotherapy, West China Hospital, Sichuan University, Chengdu, China; b AstraZeneca R&D Information China, Zhangjiang Hi-Tech Park, Shanghai, China.

**Keywords:** breast cancer, epidemiology, real-world study, survival, treatment

## Abstract

Breast cancer is a significant health issue both globally and within China. Here, we present epidemiological data for female patients diagnosed with breast cancer and treated at West China Hospital, Sichuan University, between 2005 and 2009. Patients who were diagnosed with breast cancer between 2005 and 2009 were enrolled. Data cut-off in this analysis was October 2013, allowing a minimum of 3 years’ follow-up, or follow-up until death. Data were collected and subject to statistical analyses to assess relationships between patient and cancer characteristics, treatment patterns and long-term outcomes. A total of 2252 women with breast cancer were included in the analyses. Luminal B was the most common subtype of breast cancer and human epidermal growth factor 2 (HER2)-positive (nonluminal) was the least common. Most patients had early-stage disease (stage ≤IIIa) at diagnosis. Patients with luminal A appeared to have the best overall survival (OS), compared with other subtypes. Hormone-receptor positivity was associated with improved prognosis, compared with negativity (OS hazard ratio [HR] 0.5). Late-stage compared with early-stage disease at diagnosis was associated with much poorer OS across all patients and tumor subtypes. Clear differences were apparent between breast cancer subtypes and the response to treatment. The interaction of breast cancer subtypes, treatments and disease stage is complex. One of the most important factors for improved prognosis is diagnosis and treatment at an early-stage of disease. With breast cancer becoming an increasingly important health concern, this highlights the importance of establishing systems and protocols to identify and treat patients with breast cancer as early as possible.

## Abstract 2

Supplemental Digital Content is available in the text

## 1 Introduction

Breast cancer represents a significant global health issue as one of the most common cancers. In 2012, there were nearly 190,000 new diagnoses of breast cancer in China and > 47,000 deaths from the disease.^[1]^

The phenomenon of increasing breast cancer rates in China has been acknowledged for a number of years, and is a trend that is predicted to continue into the future, with perhaps as many as 2.5 million cases of breast cancer in China by 2021.^[2,3]^ Increasing population size accounts for only a small proportion of the observed and anticipated increase in the rate of breast cancer in China, with the most important contributor being large increases in exposure to risk factors.^[4]^

With early identification and appropriate treatment, breast cancer has a high survival rate.^[5]^ Outcomes for patients with breast cancer have improved over the last few decades but there is much variation between countries. Age-standardized relative survival at 5 years ranges from >80% in Japan, Australia, North America, Finland, and Sweden to <40% in Algeria.^[6]^ Generally, survival is much lower in middle- and low-income countries, with 5-year survival ranging from <40%–60%.^[6]^

Breast cancer is a heterogeneous disease with varying clinical presentations and underlying etiologies.^[7]^ According to the 2013 St Gallen International Expert Consensus on the Primary Therapy of Early Breast Cancer, the disease can be broadly grouped into 4 intrinsic subtypes: luminal A, luminal B, human epidermal growth factor 2 (HER2)-positive (nonluminal), and triple-negative breast cancer (TNBC).^[8]^ The 4 subtypes are characterized by the presence or absence of estrogen receptor (ER) over-expression, progesterone receptor (PgR) over-expression, HER2-positivity and elevated expression of antigen Ki-67. As molecular diagnostic techniques, such as DNA and RNA microarrays, continue to evolve, further subtypes of breast cancer are expected to be identified.^[9,10]^

The national cancer registry system in China is new and not yet fully established.^[11]^ Therefore, regional cancer registries provide valuable information regarding disease epidemiology, changing rates of prevalence over time, changing behaviors with respect to treatments and patient outcomes.

West China Hospital, Sichuan University, is one of the largest hospitals in the world, with 4300 beds. Annually, there are >3 million outpatient and 150,000 inpatient visits, and >76,000 surgical procedures are performed. The hospital is ranked top for clinical research among hospitals in China. Here, we present the observed diagnostic and treatment patterns, outcomes, and factors affecting the survival of female Chinese patients diagnosed with breast cancer, patients between 2005 and 2009 and treated at West China Hospital.

## 2 Materials and methods

### 2.1 Ethical approval and consent

Ethical approval for this study was provided by the Clinical Test and Biomedical Ethics Committee of West China Hospital, Sichuan University. All patients gave their free and informed consent for their data to be collected and used for this study.

### 2.2 Patient data collection

This was an observational, population-based cohort study of female patients in Southwest China who were diagnosed with breast cancer. This study included patients with breast cancer who were treated at West China Hospital, Sichuan University, and all their data were entered into the breast cancer information management system (BCIMS). From the BCIMS records, patients were selected who received their diagnosis between 2005 and 2009, to allow follow-up for at least 3 years, or until death. The BCIMS contains patient records dating back to 1989 and records patient characteristics, medical history, breast cancer diagnosis, laboratory results, tumor pathology reports and treatments. These records were used to establish baseline diagnosis data.

### 2.3 Follow-up

Patients were followed up at least once every 4 months in the first 3 years after diagnosis. In the 3 to 5 years after diagnosis, the frequency of follow-up was reduced to once every 6 to 12 months. Annual follow-up was conducted for patients who had been diagnosed >5 years previously. Follow-up was conducted via interview at outpatient appointments or, if necessary, via telephone or postal contact. Lost to follow-up was defined as failure to make contact with the patient on >2 consecutive occasions.

### 2.4 Tumor and molecular subtype classification

Breast cancer samples were assessed by immunohistochemistry (IHC). Scoring of IHC samples was performed by board-certified pathologists in the Department of Pathology of West China Hospital using methods outlined in the American Society of Clinical Oncology and College of American Pathologists guideline recommendations.^[12]^

Samples were defined as ER or PgR-positive if at least 1% of tumor nuclei were positive for ER and/or PgR and ER/PgR-negative if <1% of tumor nuclei were positive for ER and/or PgR. Samples were categorized as negative, and + (>1%) to +++. Before 2010, testing of samples for HER2 status by fluorescence in situ hybridization (FISH) was not available at the West China hospital, samples were therefore considered to be HER2 negative if IHC scoring was zero or + and HER2-positive if IHC scoring was +++. Since 2010, 28 cases of HER2, which were categorized as positive by IHC, were screened using FISH. The “high” threshold for Ki-67 antigen was positivity in at least 14% of cells. Tumors were categorized as luminal A, luminal B, HER2, TNBC, and uncertain/unknown based on the IHC scoring (Supplemental Table S1).

The overall clinical stage of breast cancer was based on assessment of the primary tumor (T stage), the number of involved nodes (pN stage) in patients with axillary lymph node-positive breast cancer, and the presence or absence of distant metastases (M stage). Patients were further categorized as having early disease if the clinical stage was judged to be at most IIIa and late disease if the clinical stage was judged to be IIIb or IV.

### 2.5 Survival and statistical analyses

The primary endpoints of the study were overall survival (OS) and progress-free survival (PFS). The secondary endpoints metastasis-free survival (MFS) and relapse-free survival (RFS) were used to calculate PFS. A final secondary endpoint was time of survival after relapse.

Statistical analyses were performed using R version 3.2 (R Foundation for Statistical Computing, Bioconductor, http://www.bioconductor.org/). Chi-square test was conducted with built-in functions. Baseline characteristics (continuous variables) were presented as median ± standard deviation (SD). Categorical variables were compared using chi-square test and presented as percentages. Cox proportional hazards regression model and Kaplan–Meier analyses were used to determine the association between individual and multiple independent variables to patients’ outcomes. For Kaplan–Meier survival analysis, patient data were censored if OS or PFS was >8 years. Survival analyses were conducted with Bioconductor package “survcomp” (version 1.20.0, Bioconductor; http://www.bioconductor.org/) and GraphPad Prism 6 (GraphPad Software, Inc, CA). All factors with *P* < 0.05 in univariate Cox proportional hazards regression analysis were considered for multivariate analysis.

## 3 Results

### 3.1 Baseline data

#### 3.1.1 Patients

Between 2005 and 2009, a total of 2276 patients with breast cancer were entered into West China Hospital's BCIS and considered for inclusion in this cohort. Of these, 12 male patients and 12 patients with benign growths were excluded from the cohort. In total, 2252 women with breast cancer were included in these analyses (Table 1). The data cutoff for this analysis was October 2013.

**Table 1:**
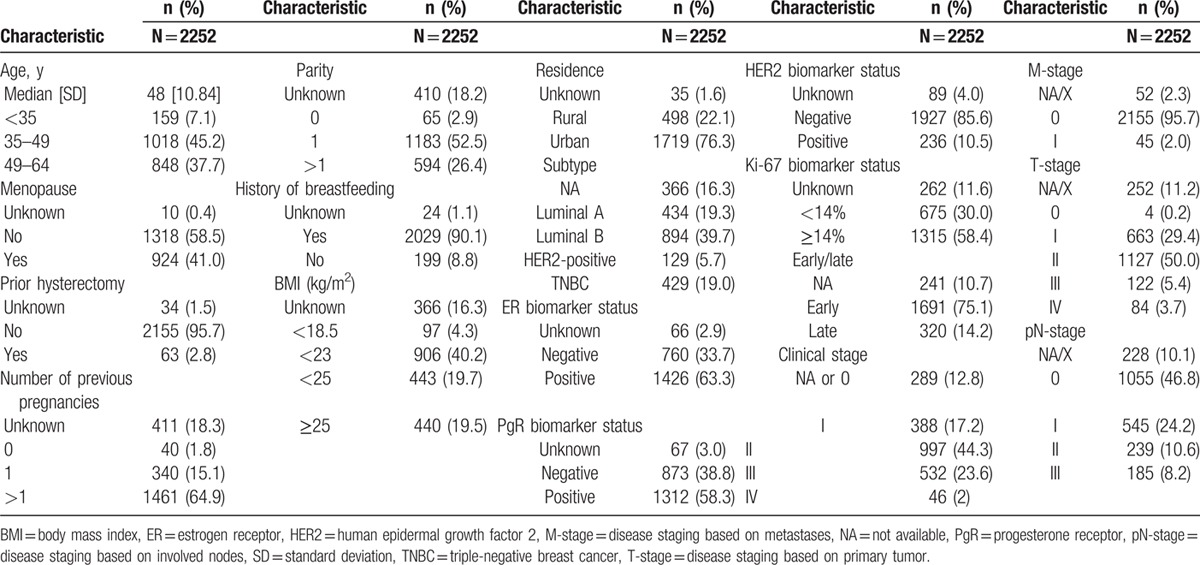
Patient and disease characteristics at diagnosis.

The median age at diagnosis was 48 years (SD = 10.84 years). Most patients were premenopausal at diagnosis (58.5%). The majority of patients had been pregnant more than once (64.9%), had a parity of at least one (78.9%), and the overwhelming majority had a history of breastfeeding (90.1%). A minority of women had undergone hysterectomy (2.8%). In addition, most women had a body mass index <25 kg/m^2^ (64.2%) and lived in urban areas (76.3%) (Table 1).

#### 3.1.2 Disease characteristics at diagnosis

The most commonly diagnosed breast cancer subtype was luminal B (n = 894, 37.7%), followed by luminal A (n = 434, 19.3%), TNBC (n = 429, 19.0%), and HER2 (n = 129, 5.7%) (Table 1). Overall, 71.9% (n = 1620) of tumors were positive for ER and/or PgR, with ER-positivity in 63.3% (n = 1426) and PgR-positivity in 58.3% (n = 1312). Most tumors (n = 1927, 85.6%) were categorized as HER2 negative (Table 1).

Most patients (n = 1691, 75.1%) had early-stage disease (stage ≤IIIa) and only a minority of patients presented with metastases (n = 45, 2.0%). With respect to tumor biology, there was a small but significant difference between the proportions of early- and late-stage disease in the patients who had PgR-positive tumors (59.7% and 52.2%, respectively; *P* = 0.01). Patients with early-stage breast cancer were significantly less likely than patients with late-stage to have tumors positive for HER2 (10.3% and 15.6%, respectively; *P* = 0.009). Similar rates of ER- and/or PgR-positivity were observed in patients with early-stage (n = 1227, 72.6%) and late-stage (n = 226, 70.6%) disease (Table 1).

#### 3.1.3 Treatment

Considering the entire cohort, most patients (n = 1519, 67.5%) received endocrine therapy (selective ER modulators [SERMs] and selective ER downregulators [SERDs] or aromatase inhibitors [AIs]). SERMs as the only endocrine therapy were received by 23.1% of patients (n = 520) and AIs as the only endocrine therapy were received by 17.1% (n = 386). Combinations of SERMs/SERDS and AIs were received by 16.7% (n = 376) of patients.

Considering patients who received anti-ER therapy, 798 had ER-positive tumors and 749 PgR-positive tumors (Table 2A). For AIs, 694 (48.7% of patients with ER-positive tumors) and 598 (45.6% of PgR-positive tumors) received AIs (Table 2A). However, the majority of patients with ER-positive tumors (n = 1291, 90.5%) did receive some form of endocrine therapy. Of the 760 patients with ER-negative tumors, 15.3% (n = 116) received anti-ER therapy and 11.3% (n = 86) received aromatase inhibitors. There were 564 patients who were both ER- and PgR-negative, of which 10.5% (n = 59) received some form of endocrine treatment.

**Table 2:**
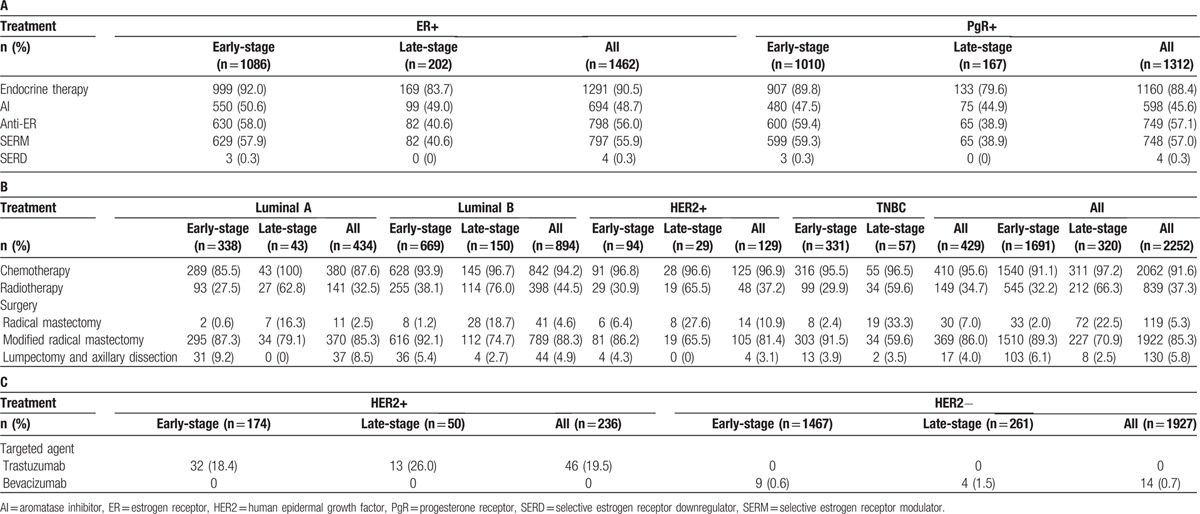
Summary of treatment for (A) ER- and PgR-positive tumors, (B) luminal A, luminal B, HER2-positive, triple-negative breast cancers, and (C) HER2-positive and HER2-negative tumors.

The vast majority of patients in our cohort had received chemotherapy (n = 2062, 91.6%). Over a third of patients (n = 839, 37.3%) received radiotherapy during their treatment course (Table 2B). Very few patients (n = 98, 4.4%) received treatment with biologic agents (Table 2C). Patients with luminal A accounted for 5.1% (5/98) of biologics use, patients with luminal B accounted for 30.6% (30/98), patients with HER2-positive accounted for 32.7 (32/98), and patients with TNBC accounted for 25.5% (25/98). Most of the luminal A patients (4/5) and the luminal B patients (27/30) received trastuzumab rather than bevacizumab. All of the HER2-positive patients received trastuzumab rather than bevacizumab. Patients with TNBC had a larger split of treatments, with most (16/25) still receiving trastuzumab but a larger proportion than seen with the other subtype (9/25) receiving bevacizumab.

There were some notable differences in treatments between patients diagnosed with early- and late-stage disease (Table 2B). Significantly more patients with early-stage than late-stage disease underwent either modified radical mastectomy (89.3% and 70.9% respectively; *P* < 0.001) or lumpectomy and axillary dissection (6.1% and 2.5%, respectively; *P* < 0.001). Just 2.0% of patients with early-stage disease underwent radical mastectomy (2.0% and 22.5%; *P* < 0.001) (Table 2B).

Patients with early-stage disease at diagnosis were significantly less likely than patients with late-stage disease to receive radiotherapy (32.2% and 66.3%, respectively; *P* < 0.001) (Table 2B). The same was true for chemotherapy, although rates were high for both patients with early-stage and late-stage disease (91.0% and 97.2%, respectively; *P* < 0.001) (Table 2B). Use of endocrine therapy for patients with ER-positive and/or PgR-positive tumors was higher for patients with early-stage disease (n = 1155, 94.1%) than for patients with late-stage disease (n = 194, 85.8%).

### 3.2 Factors affecting survival

#### 3.2.1 Patient characteristics and survival

Univariate analysis suggested that patients aged >48 years had a poorer OS than patients aged 48 years or less (hazard ratio [HR] 1.5 [95% confidence interval (CI) 1.11–1.95], *P* = 0.007). However, this finding was not confirmed in multivariate analysis. Furthermore, univariate analysis did not show any difference in OS when comparing different age brackets (≥35–<49 years, ≥49–<64 years, or ≥64 years) with patients aged <35 years as the reference.

Patients who had undergone menopause had a slightly worse OS than patients who had not reached menopause (HR 1.3 [95% CI 1.00–1.74], *P* < 0.05). Multiparity was associated with a worsening of OS (HR 2.2 [95% CI 1.17–4.19], *P* = 0.01). Patients who had been pregnant more than once had a small worsening of PFS (HR 1.8 [95% CI 1.09–2.92], *P* = 0.02).

#### 3.2.2 Tumor subtype, biomarker status, and survival

**Figure 1:**
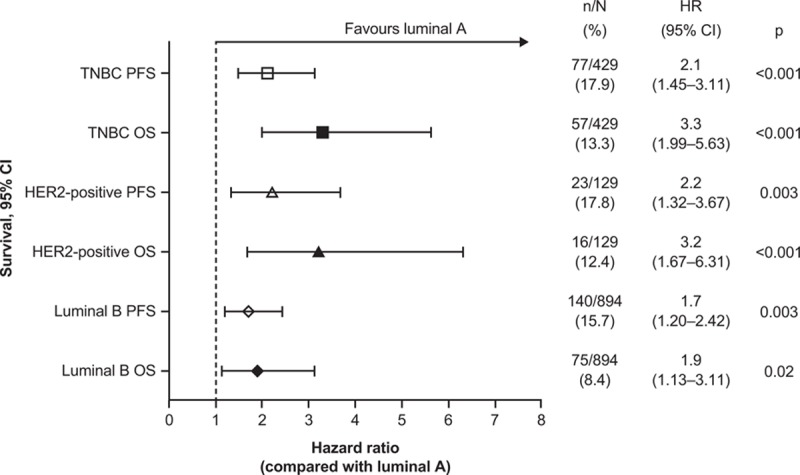
Survival differences for luminal B, HER2-positive, and TNBC subtypes compared with luminal A (followed up for at least 3 years). HER2 = human epidermal growth factor 2, n = number of events, N = number of patients at risk, OS = overall survival, PFS = progression-free survival, TNBC = triple negative breast cancer.

Univariate analysis revealed that, compared with luminal A breast cancer, patients had significantly increased HRs for OS with luminal B (HR 1.9; *P* = 0.02), HER2-positive (HR 3.2; *P* < 0.001), and TNBC (HR 3.3; *P* < 0.001) (Fig. 1). These findings were supported by Kaplan–Meier analysis for all patients (Fig. 2  A), irrespective of early- or late-stage disease (Fig. 2  B and C). A total of 13 patients had OS and PFS of 8 years or more and were censored from the Kaplan–Meier analyses.

**Figure 2:**
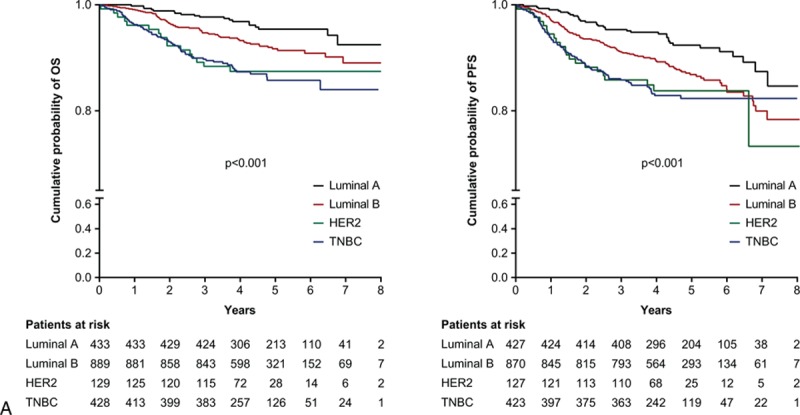
A, Kaplan–Meier analyses of OS and PFS for different breast cancer subtypes. B, Kaplan–Meier analyses of OS for different breast cancer subtypes by early- or late-stage disease. C, Kaplan–Meier analyses of PFS for different breast cancer subtypes by early- or late-stage disease. 95% CI = 95% confidence interval, HR = hazard ratio, HER2 = human epidermal growth factor 2, MFS = metastasis-free survival, OS = overall survival, PFS = progression-free survival, RFS = recurrence-free survival, TNBC = triple negative breast cancer.

**Figure 2 (Continued):**
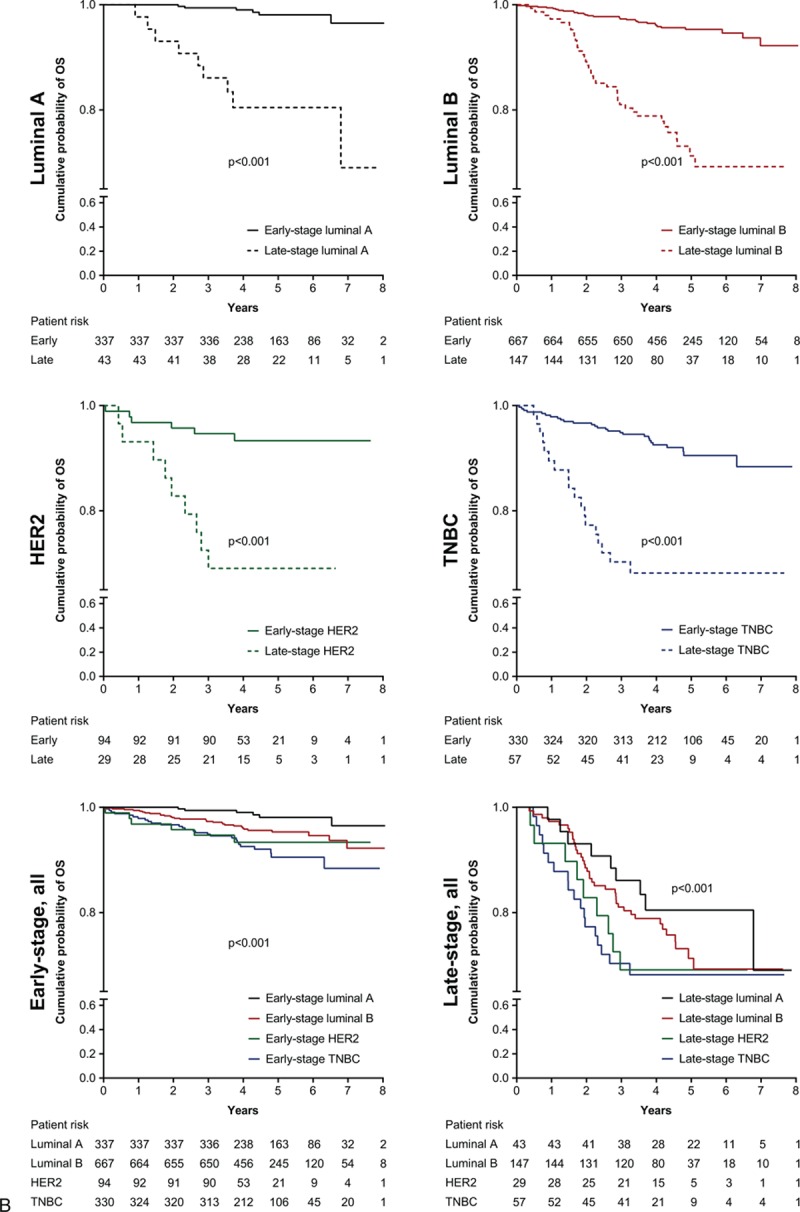
A, Kaplan–Meier analyses of OS and PFS for different breast cancer subtypes. B, Kaplan–Meier analyses of OS for different breast cancer subtypes by early- or late-stage disease. C, Kaplan–Meier analyses of PFS for different breast cancer subtypes by early- or late-stage disease. 95% CI = 95% confidence interval, HR = hazard ratio, HER2 = human epidermal growth factor 2, MFS = metastasis-free survival, OS = overall survival, PFS = progression-free survival, RFS = recurrence-free survival, TNBC = triple negative breast cancer.

**Figure 2 (Continued):**
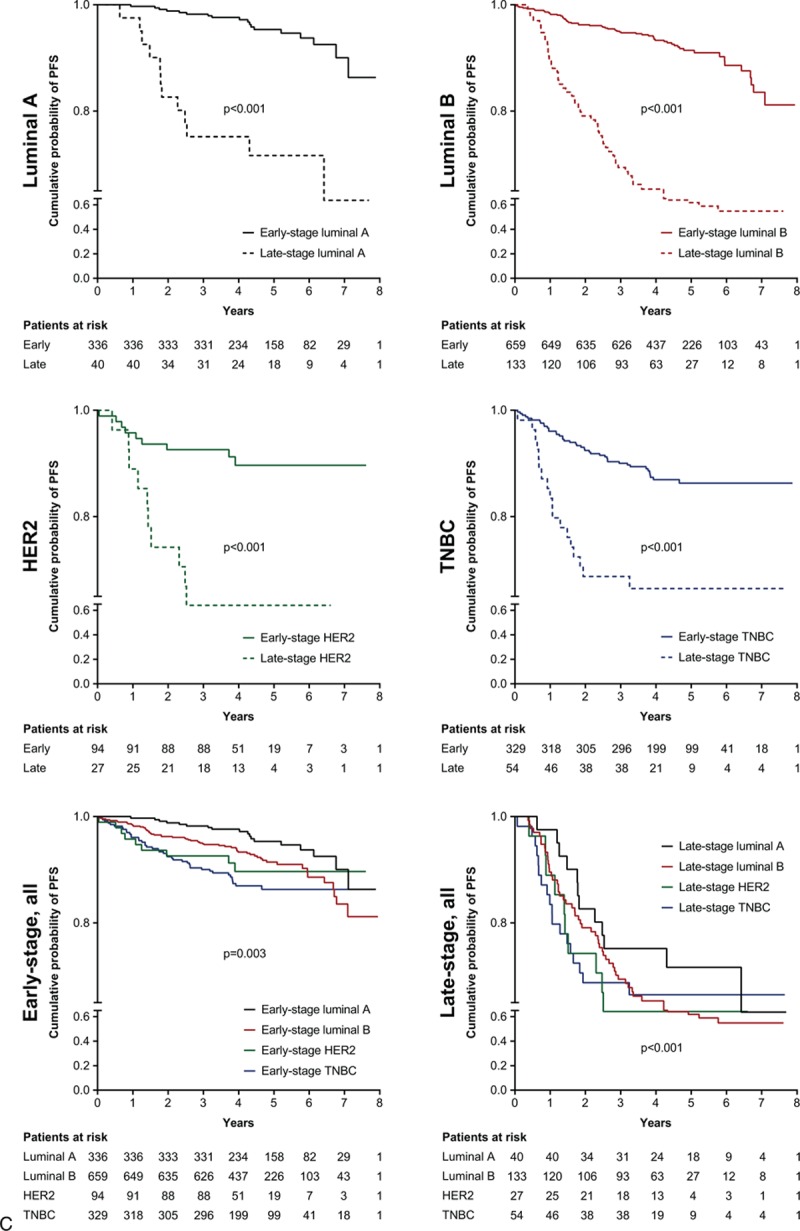
A, Kaplan–Meier analyses of OS and PFS for different breast cancer subtypes. B, Kaplan–Meier analyses of OS for different breast cancer subtypes by early- or late-stage disease. C, Kaplan–Meier analyses of PFS for different breast cancer subtypes by early- or late-stage disease. 95% CI = 95% confidence interval, HR = hazard ratio, HER2 = human epidermal growth factor 2, MFS = metastasis-free survival, OS = overall survival, PFS = progression-free survival, RFS = recurrence-free survival, TNBC = triple negative breast cancer.

Multivariate analysis also demonstrated a significantly worse prognosis for luminal B versus luminal A across all samples, with respect to OS (HR 1.8 [95% CI 1.02–3.27], *P *= 0.04) and PFS (HR 1.6 [95% CI 1.06–2.32], *P* = 0.03). However, the difference between luminal A and HER2-positive or TNBC was not significant in multivariate analysis across all samples.

Patients with ER-positive tumors had a significantly better prognosis than ER–negative patients (OS HR 0.5 [95% CI 0.35–0.61, *P* < 0.001; PFS HR 0.7 [95% CI 0.54–0.83], *P* < 0.001). The same pattern was observed for PgR-positive patients compared with PgR-negative patients (OS HR 0.5 [95% CI 0.40–0.70, *P* < 0.001; PFS HR 0.7 [95% CI 0.54–0.84], *P* < 0.001). Compared to patients who had both ER-positive and PgR-positive tumors, patients who were double-negative, ER-positive and PgR-negative or ER-negative and PgR-positive had significantly poorer OS (double-negative: HR 2.9 [95% CI 1.75–5.65], *P* < 0.001; ER-positive and PgR-negative: HR 3.1 [95% CI 1.92–10.90], *P* < 0.001; ER-negative and PgR-positive: HR 2.5 [95% CI 1.26–9.64], *P* = 0.02).

There was slight trend toward poorer OS (HR 1.4) and PFS (HR 1.2) for HER2-positive versus HER2-negative patients, but neither reached significance. Patients with high Ki-67 (≥14%) had small but significant increases in the risk of events for OS, MFS, and PFS compared with patients with low Ki-67 (<14%).

#### 3.2.3 Disease stage and survival

Every increase in T-, pN-, M-, or overall clinical stage resulted in significantly worse OS and PFS HRs (Table 3). Of particular note is the poor prognosis for clinical stage IV compared with clinical stage I, with respect to OS (HR 28.2 [95% CI 12.98–61.27], *P* < 0.001) and PFS (HR 46.0 [95% CI 26.68–79.22], *P* < 0.001).

**Table 3:**
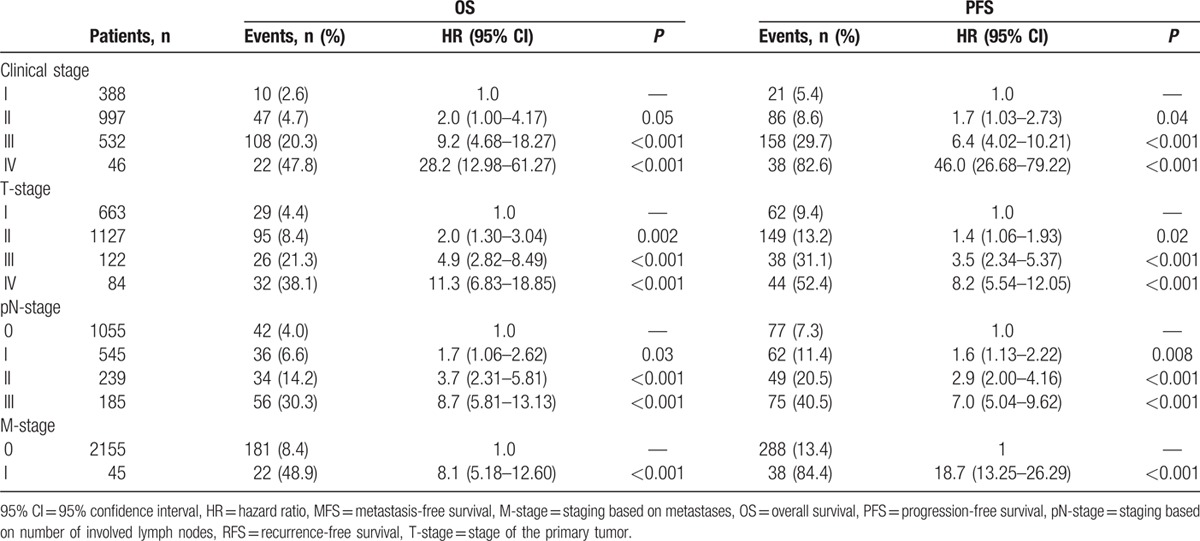
Differences in OS and PFS for patients at different disease stages.

**Figure 3:**
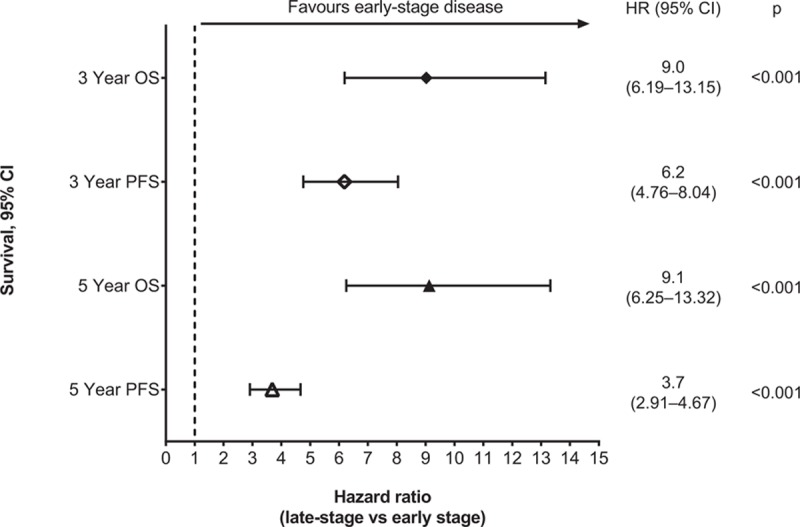
Effect of late- vs early-stage disease at diagnosis on 3- and 5-year survival (all patients). 95% CI = 95% confidence interval, MFS = metastasis-free survival, HR = hazard ratio, OS = overall survival, PFS = progression-free survival, RFS = recurrence-free survival.

Compared with patients who were diagnosed with early-stage breast cancer, late-stage diagnosis resulted in poorer OS (HR 6.7 [95% CI 5.00–8.98], *P* < 0.001) and PFS (HR 6.2 [95% CI 4.93–7.80], *P* < 0.001). Kaplan–Meier analysis also demonstrated that for each subtype, late-stage disease had significantly poorer OS and PFS than early-stage disease (Fig. 2  B and C). Multivariate analysis confirmed that late- compared with early-stage disease was associated with poorer OS across all patients (HR 6.3 [95% CI 4.38–9.13], *P* < 0.001), as well as in subtypes: luminal A (HR 30.3 [95% CI 5.98–153.41], *P* < 0.001); luminal B (HR 4.8 [95% CI 2.87–8.18], *P* < 0.001); HER2-positive (HR 13.4 [95% CI 4.07–44.33], *P* < 0.001); TNBC (HR 5.4 [95% CI 2.77–10.35], *P* < 0.001). There was a clear increase of risk for 3- and 5-year survival measures for patients with late-stage rather than early-stage disease (Fig. 3).

A total of 285 patients (12.7%) either had metastases at diagnosis and/or developed new metastases during follow-up (Supplementary Table S2). Lungs were the most common site of metastases (n = 92, 32.3%) (Supplementary Table S2). Metastasis had a large and significant impact on patient survival. In univariate analysis of all patients, patients with any metastasis had an OS HR of 56.5 (95% CI 38.27–83.34; *P* < 0.001) compared with patients without metastasis. There was an even greater impact on PFS, with an HR of 89.8 (95% CI 66.83–120.7, *P* < 0.001). With respect to sites of metastases, brain metastases appeared to have the most serious effect on prognosis, with bone as the reference site (OS HR 3.8 [95% CI 1.93–7.61], *P* < 0.001) (Supplementary Table S2).

#### 3.2.4 Treatment and survival

**Figure 4:**
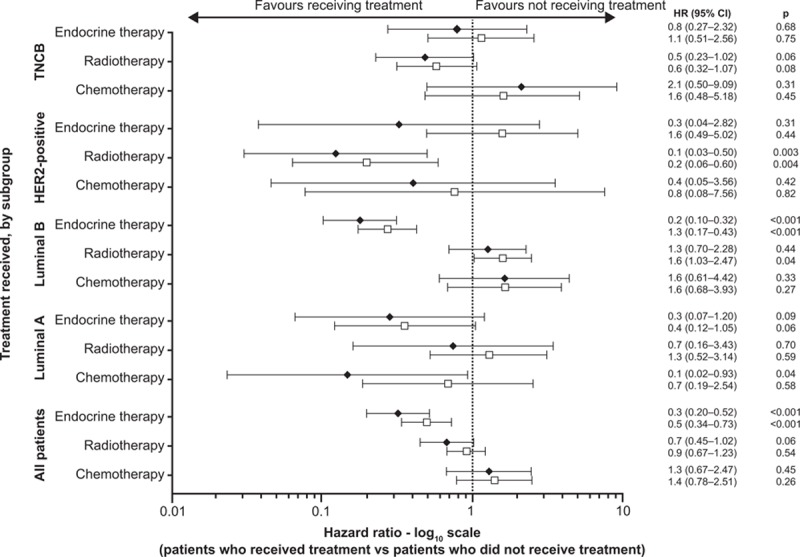
Multivariate analysis within tumor subgroups to show the effect of different treatment modalities on OS and PFS. 95% CI = 95% confidence interval, HR = hazard ratio, OS = overall survival, PFS = progression-free survival.

Within the whole population, patients who received endocrine therapy tended to do better than those who did not (HR 0.3 [95% CI 0.20–0.52], *P* < 0.001) (Fig. 4). This treatment effect was also observed in ER-positive patients (early-stage disease: HR 0.2 [95% CI 0.08–0.30], *P* < 0.001; late-stage disease: HR 0.2 [95% CI 0.09–0.27], *P* < 0.001), PgR-positive patients (early-stage disease: HR 0.1 [95% CI 0.04–0.17], *P* < 0.001; late-stage disease: HR 0.2 [95% CI 0.10–0.32], *P* < 0.001) and patients with ER-positive and/or PgR-positive tumors (early-stage disease: HR 0.2 [95% CI 0.09–0.28], *P* < 0.001; late-stage disease: HR 0.2 [95% CI 0.11–0.29], *P* < 0.001).

Luminal A patients appeared to benefit most from chemotherapy in terms of OS (Fig. 4). Patients with luminal A breast cancer were significantly more likely to receive chemotherapy as T stage, pN stage, or clinical stage increased (*P* < 0.001). Postmenopausal women with luminal A breast cancer were significantly less likely to receive chemotherapy than were premenopausal women (*P* < 0.001). Receiving either radiotherapy or endocrine therapy resulted in lower OS HRs, but did not reach statistical significance in either case (Fig. 4).

Luminal B patients had a large and significant improvement in OS and PFS for endocrine therapy versus no endocrine therapy (Fig. 4). Radiotherapy, however, was associated with a significantly poorer PFS for patients with luminal B (HR 1.6 [95% CI 1.03–2.47]; *P* = 0.04) (Fig. 4). Patients with luminal B breast cancer were more likely to receive radiotherapy with increasing T stage (*P* = 0.002), pN stage (*P* < 0.001), and clinical stage (*P* < 0.001). In addition, patients with distant metastases were more likely to receive radiotherapy than patients without metastases (*P* < 0.001). Postmenopausal patients with luminal B were significantly less likely than premenopausal patients to receive radiotherapy (*P* = 0.006).

Patients with HER2-positive breast cancer who received radiotherapy had, compared with patients who did not receive radiotherapy, a significantly improved OS (HR 0.1 [95% CI 0.03–0.50]; *P* = 0.003) and PFS (HR 0.2 [95% CI 0.06–0.60]; *P* = 0.004) (Fig. 4). Both chemotherapy and endocrine therapy appeared to show an overall trend to improving OS and PFS for HER2-positive patients, but neither reached significance (Fig. 4).

Of the 429 patients with TNBC, a small proportion received either SERMs alone (n = 16, 3.7%), AIs alone (n = 9, 2.1%), or a combination of SERMs and AIs (n = 8, 1.9%). It is unclear why these patients received these treatments and no significant associations were found between receiving these treatments and any clinical factors. There were no significant differences in OS and PFS between patients with TNBC who received endocrine therapy and those who did not (Fig. 4).

## 4 Discussion

Here, we have presented real-world data on breast cancer survival and treatment patterns from Southwest China. National cancer registries in China are not yet fully established.^[11]^ Real-world evidence such as that presented here, therefore, provide valuable insight and may be useful in identifying areas of unmet medical need and understanding the increasing burden of breast cancer in China.

The median age at diagnosis was 48 years, which was the same as that reported in previous China-wide multicenter retrospective cohort covering the period from 1999 to 2008.^[13]^ The proportion of postmenopausal patients in our cohort (41.0%) was also similar to that previously reported (37.1%).^[13]^ This suggests that the patients in our cohort were reasonably representative of other patients in China.

The most common breast cancer subtype among our patients was luminal B (39.7%), with luminal A and TNBC having very similar rates at just <20%. The least commonly observed subtype was HER2-positive, being identified in <6% of patients. This distribution is different from that previously reported in patients from Southern China, where 30.4% of patients had luminal A and 43.5% of patients had luminal B.^[14]^ A cohort of patients enrolled at Peking University Cancer Hospital >10 years (1994–2003) also showed a different distribution of cancer subtypes.^[15]^ It should be noted that the quality and availability of cytogenetic and histopathological analyses in China has been limited and is still improving. Hence, historical cohorts may not accurately represent the actual distribution of breast cancer subtypes within the population.

As techniques in China such as FISH and IHC continue to increase in availability and improve with respect to quality control, the observed distributions of breast cancer subtypes should continue to be refined. At West China Hospital, the proportion of HER2-positive breast cancers has risen from 6%, as reported in these analyses, to >26% for patients diagnosed in 2013 and 2014. This increase in accurately identifying HER2-positive breast cancer has been attributed to the introduction of FISH in 2010 and improved quality control of IHC.

In our cohort, univariate analysis demonstrated that patients with luminal A breast cancer had a significantly better prognosis than the other subtypes, with HRs ranging from 1.9 to 3.3 (*P* < 0.001–0.02). Improved survival of patients with luminal A, compared with other subtypes, has previously been reported in Chinese patients.^[14,15]^

Subanalysis by early- and late-stage disease revealed that the favorable difference in outcome of luminal A compared with the other subtypes was preserved in patients with early-stage disease. In patients with late-stage disease, the difference was no longer significant. This may be because of a universally poorer prognosis for patients with late-stage metastatic disease, regardless of subtype, leading to a lack of difference in outcomes between subtypes.

Further to this observation, multivariate analysis did not show a significant improvement in outcome for patients with luminal A, compared with HER2-positive or TNBC. This finding was unexpected but may be partly explained by the deficiencies in diagnostic techniques at the time of diagnosis, as discussed earlier. In particular, this is likely to have resulted in patients with HER2-positive tumor being mischaracterized as another subtype, such as TNBC. This subtype mischaracterization may have had a profound impact when other confounding factors, such as disease stage, were corrected for in multivariate analysis.

The majority of patients in our cohort presented with early-stage disease (75.1%) and with no metastases (87.3%). Patients who presented with late-stage disease had considerably poorer OS than patients with less advanced, early-stage disease (3-year HR 9.0, *P* < 0.001; 5-year HR 9.1, *P* < 0.001). As might be expected, late-stage disease was associated with poorer survival than patients with early-stage disease in all tumor subtypes.

Similarly, patients with metastases, of which the most common site was the lung, had considerably worse OS (HR 56.5 [95% CI 38.3–83.3], *P* < 0.001) and PFS (HR 89.8 [95% CI 66.8–120.7], *P* < 0.001) than those without. The distribution of metastases in our patients (lung [32.3%], liver [20.7%], bone [15.4%], and brain [6.7%]) was notably different from that described elsewhere in both Chinese and non-Chinese patients, particularly for bone.^[16–18]^

Although not unexpected, these results highlight the crucial importance of early detection and treatment of breast cancer. Perhaps even more so than any other variable discussed, even tumor subtype, the stage of disease is a very strong prognostic indicator. This is an area in which public health initiatives and increased awareness could help reduce the overall morbidity and mortality of breast cancer. Increasing the diagnosis of breast cancer at an early stage may improve patient outcomes and reduce the cost to the public health care systems and to patients.

Accurate identification of breast cancer subtypes is important as it informs treatment decisions and can be used to estimate overall prognosis. The optimal treatment or combination of treatments will depend on the patient, stage of disease, and the underlying biology of the tumor.^[19–21]^

We observed expected and clear treatment-effect differences across the breast cancer subtypes. In this cohort, patients with luminal B who received radiotherapy had a significantly poorer PFS than those who did not receive radiotherapy. Rather than being a shortcoming of the treatment, this is likely due to the increased likelihood of receiving radiotherapy with more clinically advanced disease. By contrast, patients with HER2-positive breast cancer had significant and notable improvements in OS and PFS when comparing those patients who received radiotherapy with those who did not. None of the treatment modalities appeared to be associated with any significant changes in outcomes for patients with TNBC. It is unclear why a small number of patients with TNBC received anti-ER therapy, AIs, or endocrine therapy. Possible explanations include mischaracterization of the tumors at treatment initiation, patients’ insistence regarding some mode of medical intervention, or clinicians deciding to try these therapies in hormone-receptor-negative patients in the hope that some therapeutic response is elicited.

There are some limitations to our study. The data were collected from a single center in 1 province of China and may not provide a representative overview of national patterns. Patient reporting can be confounded by recall-errors and misunderstanding. Only patients with access to postal facilities or telephone could be interviewed outside of appointments, which may exclude some patients in very remote regions with poor telephone or postal facilities. Medical records may have been incomplete, which may partly account for unknown or missing data relating to patients and disease baseline characteristics. Our cohort only contained small numbers of patients with metastatic disease at diagnosis or patients who experienced metastasis during follow-up. The technologies and techniques for HER2 testing were not well established in China in the 2005 to 2009 time frame, meaning that errors could have arisen from the novelty of the methods.

## 5 Conclusions

In summary, we have presented valuable breast cancer data demonstrating patient population heterogeneity, characteristics, treatment patterns, and survival in a Chinese population. The interaction of breast cancer subtypes, treatments, and disease stage is complex. In our cohort, luminal A had the most favorable outcomes, compared with the other breast cancer subtypes. One of the most important factors for improved prognosis is diagnosis and treatment at an early stage of disease. Late-stage disease is consistently and strongly associated with poorer outcomes for patients, both in terms of progression and survival. Regardless of tumor subtype, late-stage disease was the strongest prognostic marker for poor outcomes. This demonstrates that disease stage is among the most important variables for patients’ outcomes. With breast cancer becoming an increasingly important health concern, this highlights the importance of establishing systems and protocols to identify and treat patients with breast cancer as early as possible. Our data highlight the importance of tumor subtype, disease stage and presence of metastases, and the choice of appropriate adjuvant therapies on patient survival.

## Supplementary Material

Supplemental Digital Content
