# Supplementary material for: Treatment and survival patterns of Chinese patients diagnosed with breast cancer between 2005 and 2009 in Southwest China: An observational, population-based cohort study
Source: Medicine (Baltimore). 2016 Jun 24;95(25):e3865. doi: 10.1097/MD.0000000000003865 (PMC4998310; doi:10.1097/MD.0000000000003865)
Supplement: Supplemental Digital Content [file medi-95-e3865-s001.doc]

**Supplementary tables**

**Table S1. The effect of the site of metastasis on OS and PFS**

| **Luminal A** | **Luminal B** | **HER2** | **TNBC** | **Unknown** |
| --- | --- | --- | --- | --- |
| - ER- or PgR-positive - HER2-negative - Low Ki-67 | - ER-positive - HER2-negative - At least one of high Ki-67 ER-positive, HER2 negative and at least one of high Ki-67 or PgR-negative or low (<20%)   ***OR***   - ER- and HER2-positive - Any Ki-67 - Any PgR | - ER- and PgR-negative - HER2-positive | - ER-, PgR- and HER2-negative | - Pathology results unavailable or do not match the above categories |

ER, oestrogen receptor; HER2, human epidermal growth factor 2; PgR, progesterone receptor; TNBC, triple negative breast cancer

**Table S2. Criteria used to categorise tumour subtypes**

|  |  | **OS** | | | **PFS** | | |
| --- | --- | --- | --- | --- | --- | --- | --- |
|  | **Patients, n** | **Events, n (%)** | **HR (95% CI)** | **p** | **Events, n (%)** | **HR (95% CI)** | **p** |
| **Metastasis** |  |  |  |  |  |  |  |
| No | 1967 | 33 (1.7) | 1 |  | 59 (3) |  |  |
| Yes | 285 | 175 (61.4) | 56.5 (38.27–83.34) | <0.001 | 285 (100) | 89.8 (66.83–120.70) | <0.001 |
| **Metastasis location** |  |  |  |  |  |  |  |
| Bone | 44 | 17 (38.6) | 1 |  | 44 (100) |  |  |
| Brain | 19 | 16 (84.2) | 3.8 (1.93–7.61) | <0.001 | 19 (100) | 2 (1.16–3.49) | 0.01 |
| Liver | 59 | 37 (62.7) | 2 (1.10–3.48) | 0.02 | 59 (100) | 1.2 (0.78–1.73) | 0.46 |
| Lung | 92 | 57 (62) | 1.7 (1.00–2.98) | 0.05 | 92 (100) | 0.9 (0.65–1.34) | 0.71 |

95% CI, 95% confidence interval; HR, hazard ratio; OS, overall survival; PFS, progression-free survival
